# Tailoring the magnetic properties of cobalt ferrite nanoparticles using the polyol process

**DOI:** 10.3762/bjnano.10.116

**Published:** 2019-06-04

**Authors:** Malek Bibani, Romain Breitwieser, Alex Aubert, Vincent Loyau, Silvana Mercone, Souad Ammar, Fayna Mammeri

**Affiliations:** 1 Université Paris Diderot, Sorbonne Paris Cité, ITODYS UMR CNRS 7086; 2 ENS Paris Saclay, SATIE UMR CNRS 8029, 61 Avenue du Président Wilson, 94235 Cachan Cedex, France; 3 Université Paris Nord, Sorbonne Paris Cité, LSPM CNRS UPR-3407, 99, Av. J. B. Clément, 93430 Villetaneuse, France

**Keywords:** cobalt ferrite, magnetocrystalline anisotropy, magnetostriction, nanoparticle, non-stoichiometry, polyol process

## Abstract

**Background:** In extrinsically magnetoelectric materials made of two components, the direct magnetoelectric coupling arises from a mechanical strain transmission at the interface due to the shape change of the magnetostrictive component under an external magnetic field. Here, the size of the interface between the two components plays a crucial role. Therefore, the development of nanomaterials exhibiting large surface-to-volume ratios can help to respond to such a requirement. However, the magnetic nanoparticles (NPs) must be highly magnetostrictive and magnetically blocked at room temperature despite their nanometer-size. We describe here the use of the polyol process to synthesize cobalt ferrite (Co*_x_*Fe_3−_*_x_*O_4_) nanoparticles with controlled size and composition and the study of the relationship between size and composition and the magnetic behavior.

**Methods:** We used an improved synthesis of magnetostrictive Co*_x_*Fe_3−_*_x_*O_4_ NPs based on the forced hydrolysis of metallic salts in a polyol solvent, varying the fraction *x*. Stoichiometric NPs (*x* = 1) are expected to be highly magnetostrictive while the sub-stoichiometric NPs (particularly for *x* ≈ 0.7) are expected to be less magnetostrictive but to present a higher magnetocrystalline anisotropy constant, as previously observed in bulk cobalt ferrites. To control the size of the NPs, in order to overcome the superparamagnetic limit, as well as their chemical composition, in order to get the desired magnetomechanic properties, we carried out the reactions for two nominal precursor contents (*x* = 1 and 0.67), using two different solvents, i.e., triethylene glycol (TriEG) and tetraethylene glycol (TetEG), and three different durations of refluxing (3, 6 and 15 h). The structure, microstructure and composition of the resulting NPs were then investigated by using X-ray diffraction (XRD), transmission electron microscopy (TEM) and X-ray fluorescence spectroscopy (XRF), respectively. The magnetic properties were also evaluated using standard magnetometry. To measure the magnetostrictive response of the particles, the particles were sintered to dense pellets on which strain gauges were bonded, measuring the size variation radially, as a function of a dc magnetic field.

**Findings:** We found two samples, the first one being stoichiometric and magnetostrictive, and the second one being sub-stoichiometric and presenting a higher magnetization, that are appropriate to be used as ferromagnetic building blocks in nanostructured magnetoelectric materials, particularly materials based on polymers. We show that the polyol solvent and the reaction time are two key parameters to control the size and the magnetic properties of the resulting nanoparticles. We believe that these results provide relevant insights to the design of efficient magnetic and magnetostrictive nanoparticles that can be further functionalized by coupling agents, to be contacted with piezoelectric polymers.

## Introduction

Recently, extrinsically (or artificially) magnetoelectric (ME) multiferroic (MF) materials have been seriously investigated for many applications in nanoelectronics [1] and energy harvesting [2–3]. They consist of two components, one being ferromagnetic, and the other being ferroelectric. A wide range of inorganic nanostructures, defined by their connectivity, have been prepared using different synthetic approaches. Andrew et al. published a critical viewpoint paper about the current limits of such nanostructures [4]. In these materials, the ME coupling arises from a mechanical transmission of strain originating from the shape change of the magnetostrictive component under an external magnetic field, or of the piezoelectric component under an external electrical field. Thus, the geometry of the connectivity has a huge impact on the ME efficiency and high ME coefficients are expected for extrinsic multiferroics with optimized interfaces. Despites these very enthusiastic theoretical predictions, most of the experimentally measured ME coefficients appear to be significantly smaller. This discrepancy is mainly due to the difficulties in producing hybrid materials with large and perfect interfaces [5]. The use of nanomaterials exhibiting large surface-to-volume ratios instead of bulk materials can help to overcome this limitation. To the best of our knowledge, the best improvements made in this sense were those achieved by Zheng et al., who succeeded in designing self-assembled ferromagnetic CoFe_2_O_4_ nanopillars embedded in a ferroelectric BaTiO_3_ matrix [6], and by Acevedo et al. and Liu et al., who prepared CoFe_2_O_4_ and BaTiO_3_ nanoparticles (NPs) separately and co-sintered them very quickly to avoid grain growth and coarsening [7–8]. Andrew et al. also managed to optimize and maximize the hybrid interface in polymer-based multiferroics, using 10 nm magnetic nanoparticles, prepared by coprecipitation and further embedded in ferroelectric polymer fibers, made by electrospinning [9]. Focusing on this latter class of materials, the polymer exhibiting the most interesting ferro-and piezoelectric properties is a semi-crystalline fluoropolymer: poly(vinylidene fluoride) or PVDF. Mixing PVDF with magnetic nanoparticles leads to a higher polymer crystallinity, with NPs acting as nucleating points. Also, as established by Costa et al., the presence of these NPs promotes the crystallization of PVDF in its β-phase, the most electroactive one, instead of its other allotropic forms [10].

Finally, another improvement consists in making the size of the ferromagnetic component as small as possible, while maintaining an efficient strain transmission (an amplitude of ca. 30 ppm is enough for many applications [11]). Currently, the size of such nanoparticles ranges above 30 nm in diameter. Bulk single crystalline cobalt ferrite, for instance, exhibits a magnetostriction amplitude of 590 ppm [12] while its nanoparticle counterparts exhibit an amplitude between 90 and 215 ppm, depending, e.g., on their synthesis conditions and their composition [13–14]. A few years ago, Nlebedim et al. demonstrated the influence of the composition (*x*) on the magnetocrystalline anisotropy of polycrystalline Co*_x_*Fe_3−_*_x_*O_4_. The anisotropy was found to be the highest for *x* = 0.7 and 0.8 and the lowest for *x* = 0.2. However, the most interesting magnetostriction effects were found at the composition of *x* = 1. Therefore, the stoichiometry appears to be a key-parameter to tailor the magnetostrictive properties of cobalt ferrite materials [15].

Among the several chemical techniques that can be used for synthesizing magnetic metal-oxide NPs (such as thermal decomposition [16], hydrothermal method [17], co-precipitation of precursors [18], combustion reaction [19]), the polyol process has emerged as promising and versatile chemical route for the preparation of highly crystalline, monodisperse particles that are isotropic in shape [20–21]. Polyols act not only of solvents, but also as complexing ligands, avoiding the presence of any surfactant. Hydrolysis ratio, nature of polyol, synthesis temperature and precursor concentration are determining the final products in composition, shape, and size. Cobalt ferrite nanoparticles (NPs) have already been produced by the polyol process in one or in several steps. However, little research has focused on the relationship between the NP size and the magnetic properties and there is no literature at all regarding non-stoichiometric NPs. Artus et al. produced stoichiometric NPs of various sizes (from 2.4 to 6.2 nm) depending on the hydrolysis ratio, starting from iron chloride and cobalt acetate in 1,2-propane-diol [22]. The blocking temperature (*T*_B_) of the samples was found to be between 141 K (smallest NPs) and 315 K (biggest NPs). Moreover, the biggest NPs exhibited a saturation magnetization very close to that of the bulk (85 emu·g^−1^ vs 90–95 emu·g^−1^) indicating a very high crystallinity despite the small size of the NPs. Baldi et al. prepared stoichiometric NPs of different sizes, between 5 and 7 nm, in diethylene glycol, starting from iron and cobalt acetates, and using a seed-mediated growth approach [23]. They obtained monodisperse and stable particles, superparamagnetic at room temperature (RT), with, once again, high saturation magnetization values for the largest ones. Hyeon et al. succeeded to produce cobalt ferrite NPs of 12 nm in diameter and evidenced a blocked ferromagnetic behavior for these particles at RT (*T*_B_ = 320 K) [24]. They also used an etherdiol solvent as polyol during moderate heating.

Based on these former studies, sizes larger than 10–12 nm are necessary if one wants to obtain blocked cobalt ferrite particles at room temperature (*T*_B_ > RT) [25]. At the same time, the size must be as small as possible to extend the hybrid interface in the further nanostructured hybrid ME materials and to optimize the strain transmission as well as the ME coupling.

Here, we aim to control the size of the NPs through the choice of the solvent, triethylene glycol (TriEG) and tetraethylene glycol (TetEG) with different boiling temperatures (*T*_b_ = 285 and 325 °C, respectively, for TriEG and TetEG) and through the refluxing time (from 3 to 15 h), assuming that a higher reaction temperature and longer reaction times will yield larger particles. In addition, we will examine different chemical compositions of the particles, i.e., the stoichiometric composition (*x* = 1) and the non-stoichiometric composition (*x* = 0.67), expecting a higher magnetostrictive coefficient for the former and a higher magnetocrystalline energy constant for the latter.

## Results and Discussion

### Structural characterization of the Co*_x_*Fe_3−_*_x_*O_4_ nanoparticles

Nine samples have been prepared. They consist of Co*_x_*Fe_3−_*_x_*O_4_ nanoparticles distributed in two series: six of them are stoichiometric (*x* = 1) and the three others are sub-stoichiometric in cobalt (*x* = 0.67). For the first series (*x* = 1), triethylene glycol (TriEG) and tetraethylene glycol (TetEG) polyol have been used as solvents and the reaction was carried out over three different periods of time (3, 6 and 15 h). For the second series (*x* = 0.67), two attempts have been made in TriEG for 3 and 6 h, and only one in TetEG for 3 h. The main features of all prepared compositions are collected in Table 1.

**Table 1:** Main structural features of the Co*_x_*Fe_3−_*_x_*O_4_ nanoparticles, prepared by the polyol process.

sample	(*x*)	polyol	reaction time	*d* (nm) from XRD	*d* (nm) from TEM	cell parameter (Å) from XRD

Co-1-TriEG-3	1	TriEG	3	7 ± 1	5.6 ± 0.2	8.404 ± 0.002
Co-1-TriEG-6	1	TriEG	6	8 ± 1	6.7 ± 0.2	8.401 ± 0.002
Co-1-TriEG-15	1	TriEG	15	9 ± 1	8.4 ± 0.2	8.402 ± 0.002
Co-1-TetEG-3	1	TetEG	3	8 ± 1	7.8 ± 0.3	8.405 ± 0.002
Co-1-TetEG-6	1	TetEG	6	10 ± 1	9.6 ± 0.2	8.404 ± 0.002
Co-1-TetEG-15^a^	1	TetEG	15	13 ± 1	12.0 ± 0.3	8.399 ± 0.002

Co-0.67-TriEG-3	0.67	TriEG	3	12 ± 1	10.2 ± 0.2	8.397 ± 0.002
Co-0.67-TriEG-6	0.67	TriEG	6	13 ± 1	12.1 ± 0.2	8.397 ± 0.002
Co-0.67-TetEG-3	0.67	TetEG	3	13 ± 1	12.0 ± 0.3	8.398 ± 0.002

^a^Co-1-TetEG-15 shows traces of metallic Co.

**Figure 1:**
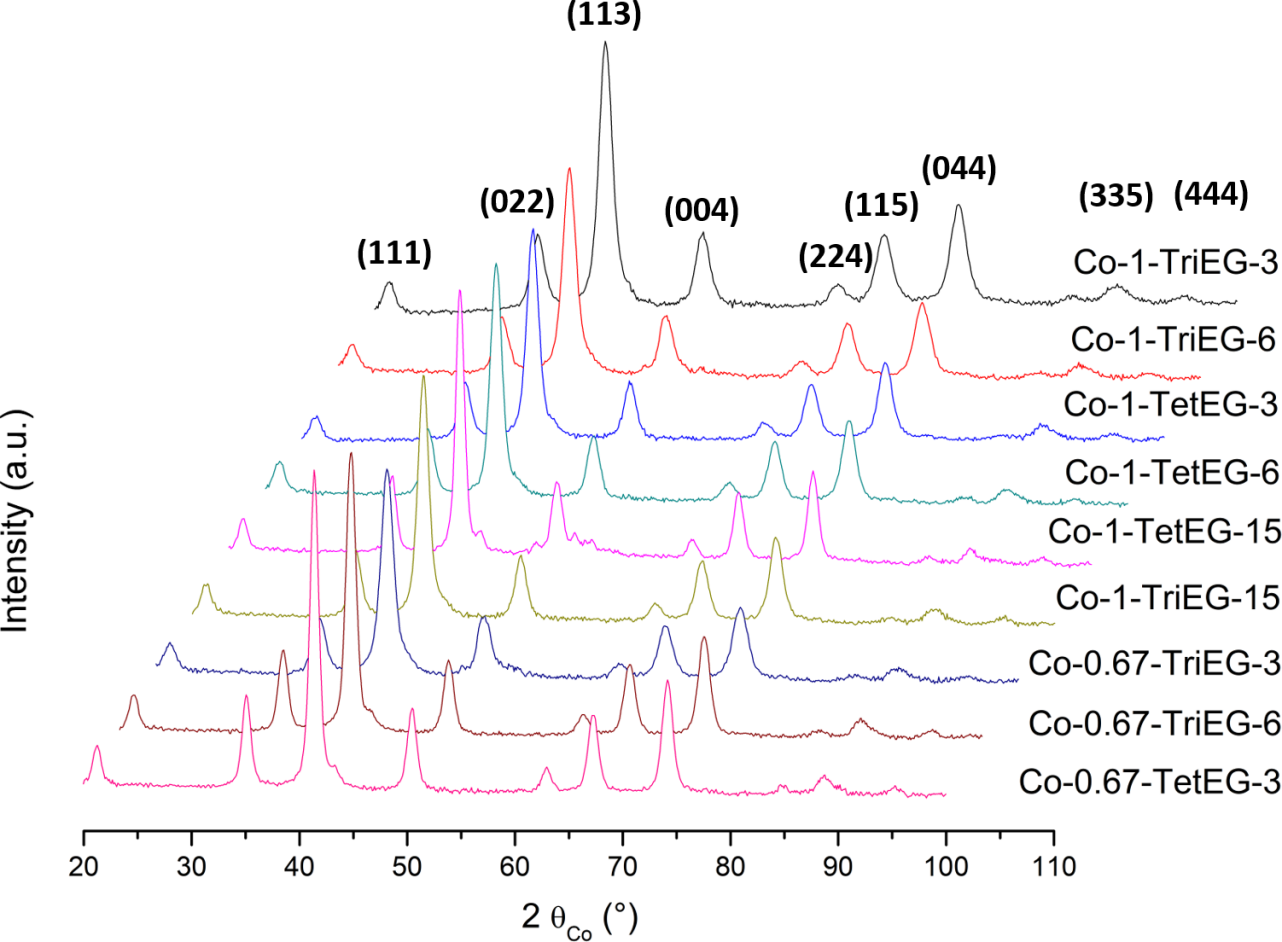
XRD patterns of all the produced Co*_x_*Fe_3−_*_x_*O_4_ powders.

**Figure 2:**
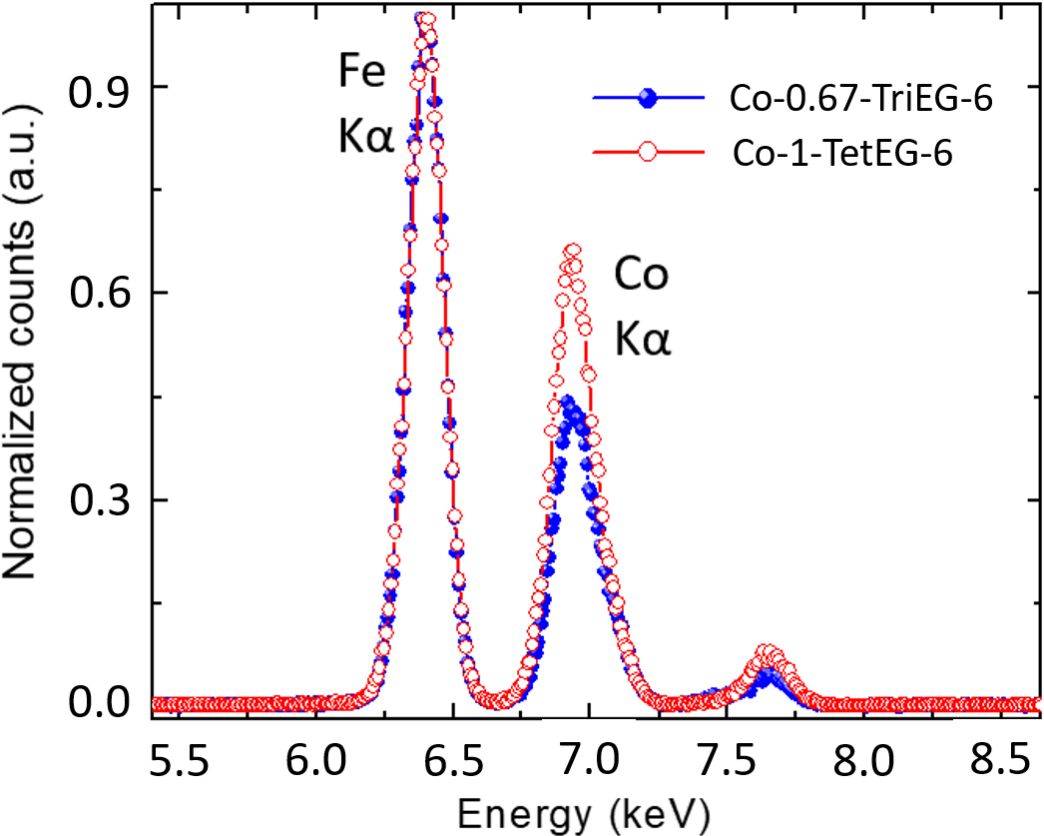
X-ray fluorescence experiments performed on two representative samples, Co-1-TetEG-6 and Co-0.67-TriEG-6.

We have recorded the X-ray diffraction (XRD) patterns of all cobalt ferrite samples (Figure 1). They are all matching very well with the cubic spinel structure (ICDD no. 98-003-9131). The crystal size of each sample has been estimated through computational Rietveld refinements using MAUD software [26] (Table 1). Then, the compositions have been checked by X-ray fluorescence (XRF) experiments (Figure 2).

**Figure 3:**
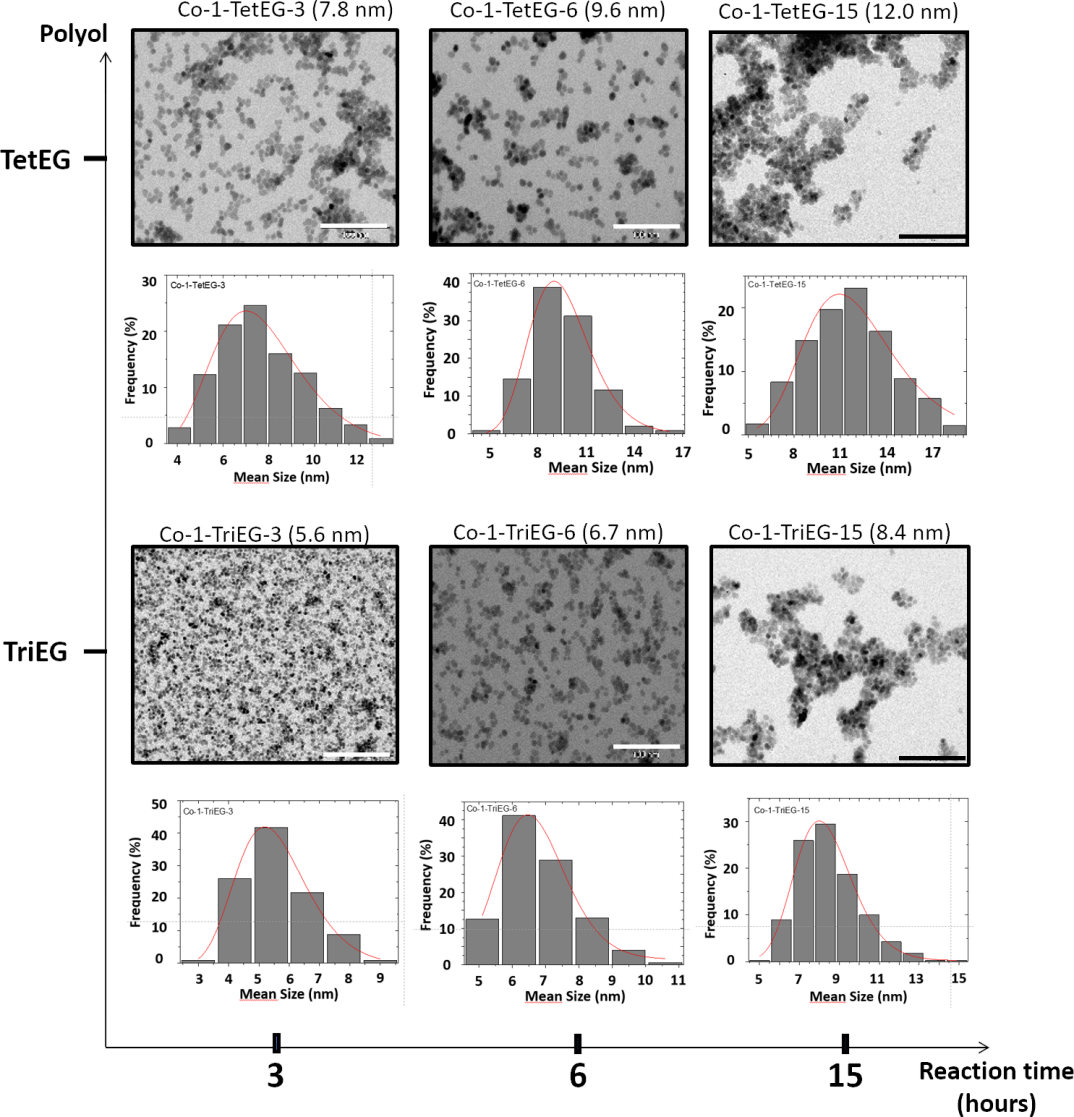
TEM images of CoFe_2_O_4_ NPs as a function of the polyol nature and the reaction time, and the corresponding diameter distributions and log-normal fits. Scale bar = 100 nm.

**Figure 4:**
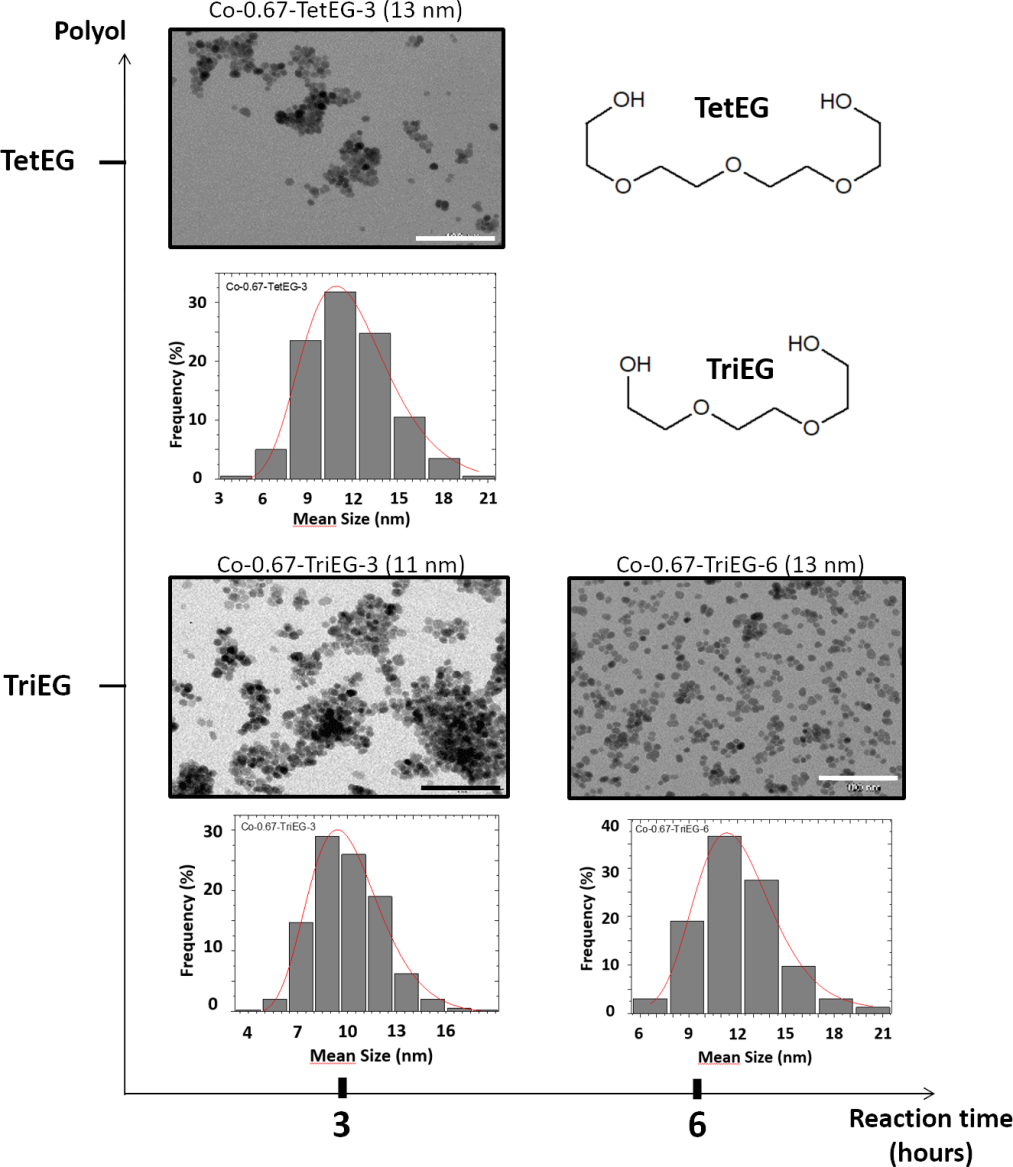
TEM images of Co_0.67_Fe_2.33_O_4_ NPs as a function of the polyol nature and the reaction time, and the corresponding diameter distributions and log-normal fits. Scale bar = 100 nm. The formulas of the polyols are given in the insert.

The crystal sizes estimated from the XRD patterns are in good agreement with the mean diameters deduced from transmission electron microscopy (TEM) images (Table 1), meaning that the nanoparticles are monocrystalline. The micrographs given in Figure 3 and Figure 4 present the two series of nanoparticles (*x* = 1 and *x* = 0.67) as a function of the polyol and as a function of the reaction time. All of them appear to be quite uniform in size since the standard deviations do not exceed 20% of the average diameters. The particle size histograms presented in Figure 3 and Figure 4 have been made applying Sturges’ rule [27]. They were then fitted using a log-normal function (Equation 1) and the median diameter *D* as well as the dispersion σ were determined (see Table 1).

[1] $$f\left(D\right)=\frac{1}{\sqrt{2\pi \sigma D}}\times \exp\left[\frac{\ln^{2}\left(\frac{D}{D_{0}}\right)}{2\sigma^{2}}\right]$$

Then, the mean diameter <*D*> and standard deviation σ_D_ were calculated (Equation 2 and Equation 3).

[2] $$\langle D\rangle \;=\;D_{0}\times \exp\left(\frac{\sigma^{2}}{2}\right)$$

[3] $$\sigma_{D}=\;\langle D\rangle \times \;{\left[\exp\left(\sigma^{2}-1\right)\right]}^{1/2}$$

### Influence of the time of synthesis

The reactions were carried out for different periods of time: 3, 6 and 15 h. Table 1 shows unambiguously that the NP diameter is increasing when the duration of the reaction increases, when the other conditions are the same.

### Influence of the solvent

We used two polyols: TriEG and TetEG (Figure 4). The former presents a shorter backbone and a higher dielectric constant (ε_r(TriEG)_ = 23.7 vs ε_r(TetEG)_ = 20.4) while the latter exhibits a larger molecular weight and is assumed to be a little more polar (µ_TetEG_ = 5.84 *D* vs µ_TriEG_ = 5.58 *D*) [28]. Regardless of reaction time and composition, we observe that the NP diameter is higher when tetraethylene glycol is used instead of triethylene glycol. Dipolar moment and dielectric constant of the two molecules are very similar and we can assume that they both exhibit the same strength to dissolve the ionic precursors. TetEG has a longer backbone than TriEG and can chelate bigger colloidal species, which may promote the growth better than TriEG. Another parameter may contribute to explain this size difference between the TriEG- and the TetEG-derived particles, i.e., the reaction temperature and the boiling points of the polyols. Indeed, the refluxing temperature was observed to be lower for TriEG but not so much regarding the boiling points of the two polyols. Considering the very probable hypothesis that particle nucleation proceeds when the boiling temperature of the reaction medium is reached (leading to the lowest viscosity), one can expect the formation of much more nuclei when the reaction temperature is close to the boiling point of the solvent [29]. This was the case when TriEG was used. Thus, the crystal growth by solute diffusion occurred on a larger number of nuclei, leading to a smaller final particle size: In contrast, a smaller number of nuclei was produced in TetEG, since the reaction temperature was considerably lower than the boiling point.

### Influence of the starting stoichiometry

Two chemical compositions of Co*_x_*Fe_3−_*_x_*O_4_ NPs have been prepared: *x* = 1 and *x* = 0.67. We observe that, for the same polyol used and the same time of reaction, the sub-stoichiometric nanoparticles are always bigger by at least 5 nm than the stoichiometric ones. The dependence of the NP size on *x* is still poorly understood, and it would be interesting to investigate it. But at this stage of our study we only noticed it, with the aim of elucidating it in further experiments.

### Aggregation

From the XRD and TEM measurements, we have deduced the average diameter of the produced particles, assuming them to be almost spherical single crystals uniform in size. Moreover, from TEM images, we can evaluate the morphology developed by the NPs. In the stoichiometric samples, the nanoparticles obtained after 15 h of reaction are clearly more aggregated than those obtained after 6 h, most likely due to stronger van der Waals and/or magnetostatic interactions between bigger nanoparticles (promoted by the drying of the NPs during the sample preparation for TEM) resulting in the clustering of particles. This observation has been made after syntheses with TetEG and TriEG, and after drop casting the same quantity of NPs under the same conditions. In the sub-stoichiometric series, Co-0.67-TriEG-3 NPs exhibit the highest degree of aggregation.

### Magnetic properties

Standard magnetometry has been carried out on all Co*_x_*Fe_3−_*_x_*O_4_ NPs with a special emphasis on the biggest NPs, for which a blocked ferromagnetic behavior is expected at RT (*T*_B_ > RT).

**Figure 5:**
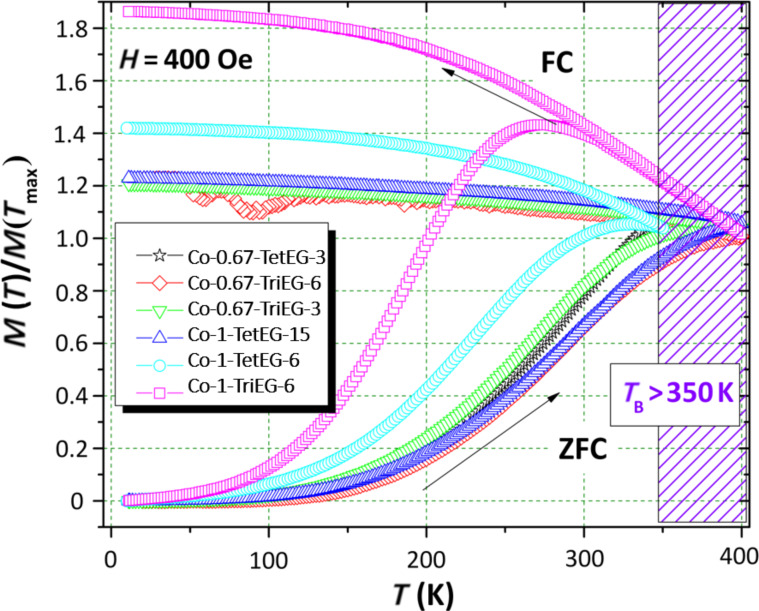
Thermal variation of the normalized DC magnetic magnetization measured in ZFC and FC conditions.

**Figure 6:**
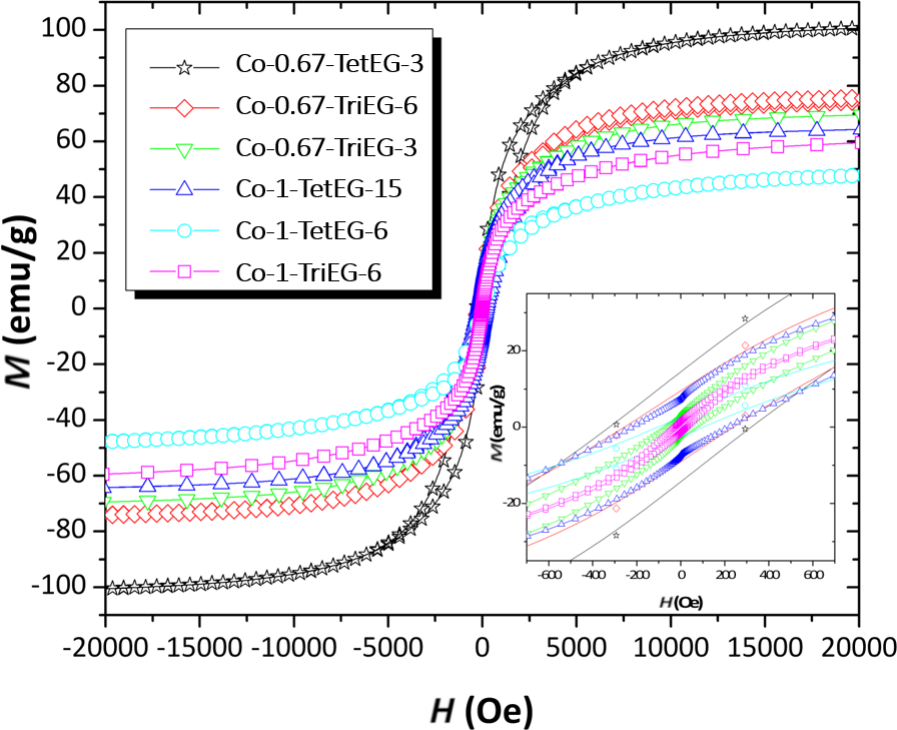
Magnetisation curves of NPs measured at 300 K. Inset: zoom-in of the coercive behavior.

The zero-field-cooled (ZFC) and field-cooled (FC) magnetization as a function of the temperature is shown in Figure 5. In general, the recorded magnetic behavior is that of ferrite particles in their single magnetic domain state. As it is summarized in Table 2, quite all the samples showed very high *T*_B_ values (>300 K). The only superparamagnetic nanoparticles at room temperature are the stoichiometric particles synthesized in TriEG for 6 h (Co-1-TriEG-6). In this case, *T*_B_ was found to be equal to 275 K; although the saturation magnetization value is pretty good for this composition at the nanoscale level (*d* = 8 nm), the superparamagnetic behavior observed at room temperature is not suitable for the targeted applications. Interestingly, such high *T*_B_ values confirm the high crystalline quality of the produced NPs. We pursued our investigations by plotting the variation of the magnetization of these particles as a function of the magnetic field at RT, typically *T* = 300 K (Figure 6). Low-temperature (*T* = 10 K) hysteresis behavior is not reported, as it is comparable to that at the RT; however, the coercive fields that have been measured at this temperature are summarized in Table 2, as well as those obtained at RT.

**Table 2:** Blocking temperature (*T*_B_), saturation magnetization (*M*_S_) and coercive field (*H*_C_) of the Co*_x_*Fe_3−_*_x_*O_4_ nanoparticles.

sample	*T*_B_ (K)	*M*_S_ (emu·g^−1^) at 300 K	*H*_C_ (Oe) at 300 K	*H*_C_ (Oe) at 10 K

Co-1-TriEG-6	ca. 275	62	28	690
Co-1-TetEG-6	ca. 330	51	94	10041
Co-1-TetEG-15^a^	>350	67	220	13300
Co-0.67-TriEG-3	>350	71	60	8000
Co-0.67-TriEG-6	>350	77	220	780
Co-0.67-TetEG-3	>350	104	300	13700

^a^Co-1-TetEG-15 shows traces of metallic Co.

Higher values of coercivity have been observed for NPs synthesized in TetEG. Again, extending the reaction time in TriEG up to 6 h does not yield the high coercivity observed in TetEG at 300 and 10 K. Thus, for equal nanoparticle sizes, equal time of reaction and equal composition, the TetEG solvent seems to optimize the magnetic behavior of the nanopowders in regard to the targeted applications. Additionally, they present saturation magnetization values among the highest that can be found in the literature for this particle size [30–31].

**Figure 7:**
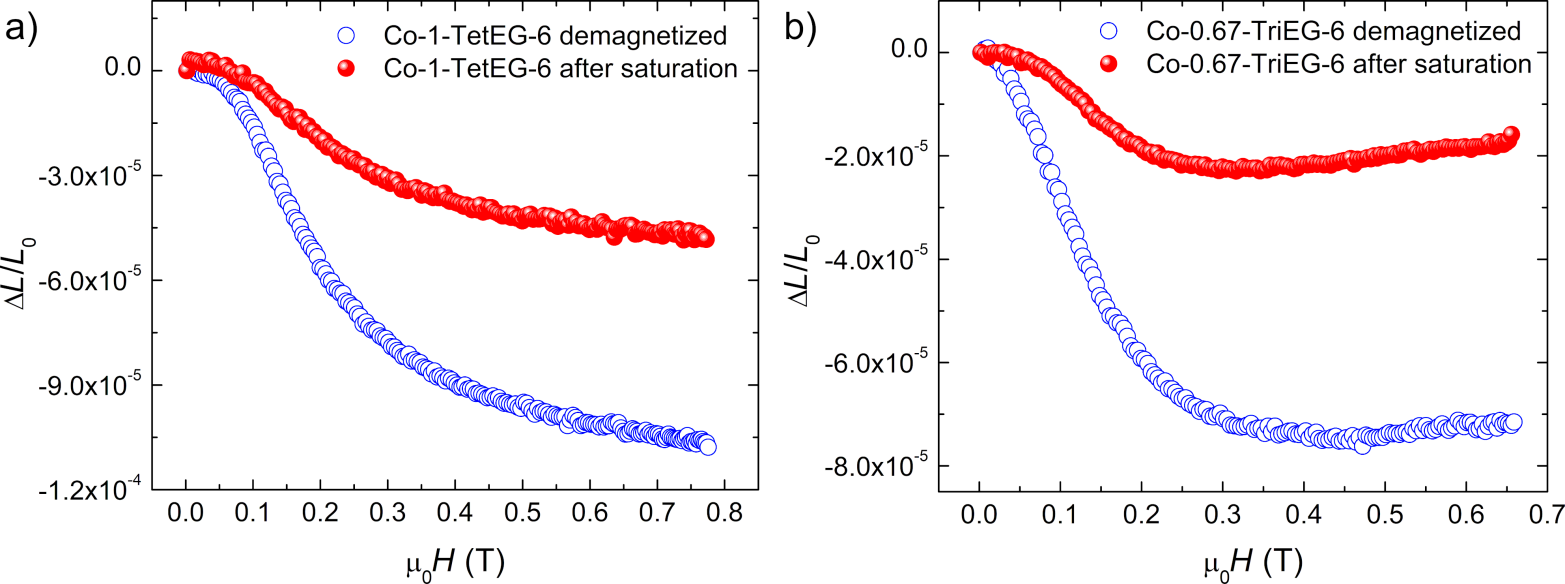
Radial magnetostriction as a function of the applied magnetic field for a) Co-1-TetEG-6 and b) Co-0.67-TriEG-6 consolidated derivatives, with measurements carried out from either the demagnetized (blue circles) or the in-plane saturated (red circles) state.

Based on all these structural and magnetic results, we chose to focus on the following two samples: Co-1-TetEG-6 and Co-0.67-TriEG-6. As magnetostriction measurement requires bulk samples, the samples were sintered by using spark plasma sintering. We are aware that the measured magnetostrictive coefficients on the prepared pellets do not correspond exactly to those of the bare particles, but they are quite indicative of the magnetostrictive behavior of the starting powders. Magnetostriction measurements were performed by applying an in-plane external magnetic field (see the Experimental section). The results are presented in Figure 7.

For the Co-0.67-TriEG-6 consolidated derivative, a maximal radial magnetostrictive deformation (*L* − *L*_0_)/*L*_0_ of −76 ppm has been observed for the demagnetized state, when the magnetic field is applied for the first time (*L*_0_ is the initial length of the material with *H* = 0). After the in-plane saturation of the magnetization, a second measurement has been carried out showing a Δ*L*/*L*_0_ coefficient strongly reduced to −23 ppm due to the remanence effect of the magnetization. To recover the magnetostriction measured during the first cycle, it is necessary to preliminary demagnetize the sample or to saturate the magnetization along a transversal direction.

For the Co-1-TetEG-6 consolidated derivative, the highest magnetostrictive coefficient of −106 ppm has been obtained from the demagnetized state while the second cycle of measurements indicated a deformation of −47 ppm. We therefore observed that the magnetic history has similar effects on the magnetostrictive response of both samples. Moreover, reducing the Co amount in the initial powders by ca. 30% leads to a loss of ca. 30% of the magnetostrictive response. Such a result is quite reasonable since the magnetostriction of cobalt ferrites is induced by Co^2+^ ions in octahedral sites and the spin–orbit–lattice interaction with the distorted cubic crystal field. The other striking conclusion is that the larger deformation of stoichiometric cobalt ferrites nanoparticles under magnetic field makes them more promising to enhance the ME properties of nanomaterials.

## Conclusion

We have described here the synthesis procedures of magnetostrictive Co*_x_*Fe_3−_*_x_*O_4_ (*x* = 1 and *x* = 0.67) NPs, using the polyol process. The produced NPs have been well characterized using X-ray techniques (diffraction and fluorescence) and transmission electron microscopy. Most of the NPs are above 10 nm in size, magnetically stable and blocked at room temperature (*T*_B_ > RT). Moreover, they exhibit saturation magnetization values among the highest presented in literature for their typical size. For the syntheses, we used two different polyol solvents, TriEG and TetEG and carried out the reactions for three different periods of time (3, 6 and 15 h). We could identify two samples, the first one being stoichiometric and the second being sub-stoichiometric, appropriate for the use as ferromagnetic building blocks in nanostructured magnetoelectric materials, particularly polymer-based hybrid materials. We hope this work is providing some insight into the ability to design efficient magnetic and magnetostrictive particles that can be further functionalized by coupling agents such as phosphonic acids to be introduced in polymers [32–33].

## Experimental

### Synthesis of the nanoparticles

The synthesis of the Co*_x_*Fe_3−_*_x_*O_4_ nanoparticles (NPs) was carried out using the polyol process [22], starting from iron and cobalt acetates, Fe(CH_3_COO)_2_ and Co(CH_3_COO)_2_·4H_2_O (Acros and Aldrich, respectively) in two nominal ratios (*x* = 1 or *x* = 0.67) [34]. We used two different polyols, TriEG and TetEG. The reaction mixture was heated up to reflux (270 or 290 °C, depending on the solvent) and maintained under reflux for 3, 6 or 15 h to obtain single-phase NPs of various sizes. After being cooled to RT, the black nanoparticle powders were recovered by several centrifugation cycles and washing with acetone. At the end, they were dried overnight in air at 50 °C. Table 1 is collecting all the main features of these samples.

### Characterization of the nanoparticles

#### Structure

The XRD patterns of the recovered powders have been recorded on an X’Pert Pro PANanalytical diffractometer (Co Kα radiation), in the range of 10–100° (2θ) with a scan step of 0.02°. The morphology of the NPs has been determined by TEM observations, using a JEOL-100 CX II microscope operating at 100 kV. The mean diameter and standard deviation were inferred from image analysis of ca. 350–400 particles using ImageJ software and correlated to the microstructural information (mainly crystal size and micro-strain-induced lattice deformation) inferred from Rietveld analysis of the XRD data using MAUD software [26]. The chemical analysis of the particles was checked by XRF, using a Panalytical MINIPAL4 X-ray fluorescence spectrometer, equipped with a rhodium X-ray tube operating at 30 kV and 87 μA current emission. Quantification was determined from pre-plotted calibration curves using standard Co and Fe solutions.

#### Magnetic properties

Direct-current magnetic measurements were performed using a Quantum Design MPMS 3 superconducting quantum interference device working as a vibrational standard magnetometer. The thermal variation of the magnetic susceptibility χ(*T*) were recorded in both ZFC and FC modes, in the temperature range of 10–400 K under a magnetic field of 400 Oe. The magnetization as a function of the magnetic field *M*(*H*) was also recorded at low temperature (10 K) and room temperature (300 K) cycling the magnetic field between −70 kOe and +70 kOe. A sampling tube made from a specific pod from Quantum Design has been used to mechanically block the analyzed powders (few milligrams) during the measurements.

#### Magnetostriction

**Figure 8:**
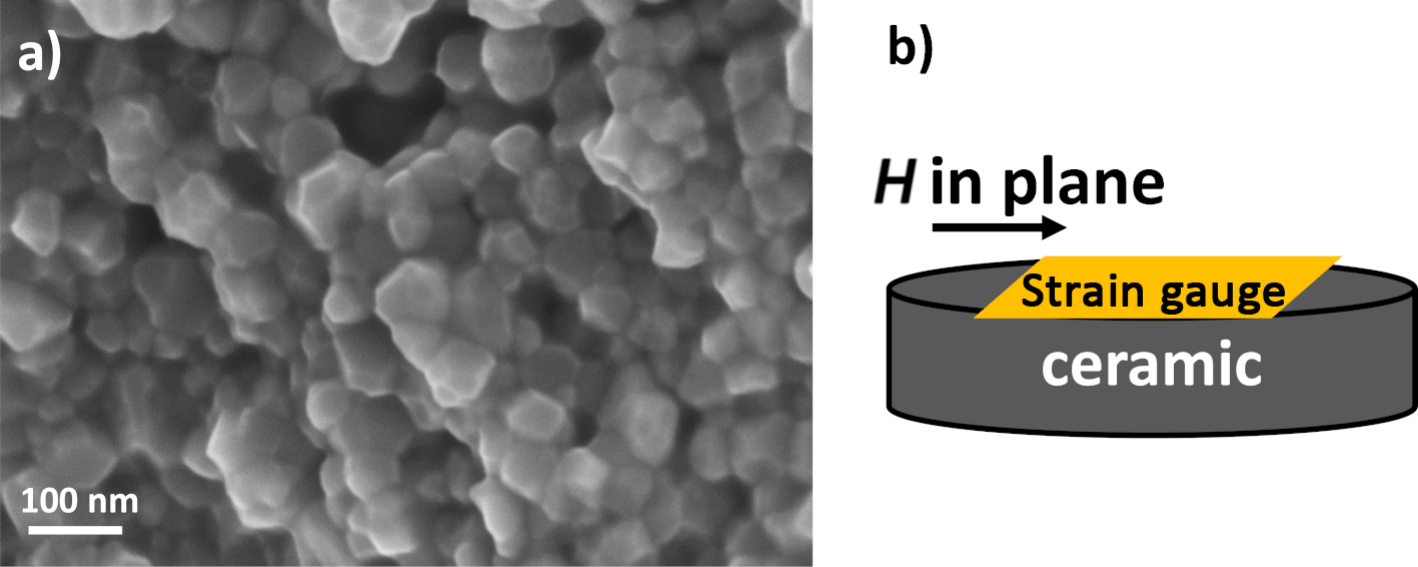
a) Representative scanning electron microscopy (SEM) image of the cobalt ferrite consolidated derivative and b) a schematic illustration of the custom-made magnetostriction measurement setup.

Selected particles have been first consolidated into dense pellets by using spark plasma sintering, applying a uniaxial pressure of 100 MPa and heating the pressed powder up to 500 °C in a graphite die (more details are given in [35]). Then, a resistive strain gauge (EA-06-062TT-120, Micro-Measurements) was glued (with epoxy resin) on the top face of each polished pellet, to perform extensometry measurements in presence of a longitudinally applied magnetic field *H* (Figure 8b). The in-plane direction of *H* has been chosen to avoid demagnetizing field effects occurring in the out-of-plane direction, which is known to affect the ME response. Then, the relative strain (*L* − *L*_0_)/*L*_0_ of each pellet was deduced from the measurement of the resistance relative variation (*R* − *R*_0_)/*R*_0_ of the gauge following: (*L* − *L*_0_)/*L* = (1/*K*). (*R* − *R*_0_)/*R*_0_, where *K* is the gauge factor (*K* = 2), *R*_0_ = 120 Ω is the initial unstrained resistance of the gauge, and *L*_0_ is the unstrained length of the active part of the gauge. The sample has been placed into an electromagnet, and a magnetic field varying from 0 up to 1 T has been applied. This is sufficient to reach the Co*_x_*Fe_3−_*_x_*O_4_ saturation magnetization (generally, µ_0_*H*_sat_ < 0.5 T at RT). In our measurements, (*L* − *L*_0_)/*L* coincides with the magnetostriction coefficient λ_11_ because the strain measurement is done along the direction of the applied field *H*. For magnetoelectric applications, one should note that the transverse magnetostrictive coefficient (λ_21_) is also of interest. In case of isotropic samples, the transversal coefficient is expected to be half the longitudinal one and opposite in sign [36].

Using this procedure, we have evaluated the magnetostrictive properties of the two most interesting samples, i.e., Co_0.67_Fe_2.33_O_4_, known to exhibit the highest magnetocrystalline anisotropy (Co-0.67-TriEG-6), and CoFe_2_O_4_, known to present the highest magnetostriction (Co-1-TetEG-6).
